# Decreased T cell reactivity to Epstein–Barr virus infected lymphoblastoid cell lines in multiple sclerosis

**DOI:** 10.1136/jnnp.2008.161018

**Published:** 2008-11-17

**Authors:** M P Pender, P A Csurhes, A Lenarczyk, C M M Pfluger, S R Burrows

**Affiliations:** 1 The University of Queensland, School of Medicine, Queensland, Australia; 2 Department of Neurology, Royal Brisbane and Women’s Hospital, Queensland, Australia; 3 The University of Queensland Centre for Clinical Research, Queensland, Australia; 4 Queensland Institute of Medical Research, Queensland, Australia

## Abstract

**Objective:**

To investigate T cell and antibody immunity to Epstein–Barr virus (EBV) in multiple sclerosis (MS).

**Methods:**

Immunoglobulin G (IgG) immunity to EBV nuclear antigen 1 (EBNA1) and viral capsid antigen was measured by enzyme linked immunosorbent assays, and T cell immunity was assessed using enzyme linked immunospot assays to measure the frequency of peripheral blood mononuclear cells (PBMC) producing interferon γ in response to autologous EBV infected B cell lymphoblastoid cell lines (LCL) in 34 EBV seropositive healthy subjects and 34 EBV seropositive patients with MS who had not received immunomodulatory therapy in the previous 3 months.

**Results:**

Patients with MS had increased levels of anti-EBNA1 IgG but a decreased frequency of LCL specific T cells compared with healthy subjects. Using purified populations of CD4^+^ T cells and CD8^+^ T cells, we showed that the LCL specific response resides predominantly in the CD8^+^ population, with a frequency 5–7-fold higher than in the CD4^+^ population. The decreased CD8^+^ T cell response to LCL in MS was not caused by decreased HLA class I expression by LCL, and LCL from MS patients could be killed normally by HLA matched EBV specific cytotoxic CD8^+^ T cell clones from healthy subjects. Furthermore, the decreased CD8^+^ T cell immunity to EBV was not due to a primary defect in the function of CD8^+^ T cells because EBV specific cytotoxic CD8^+^ T cell lines could be generated normally from the PBMC of patients with MS.

**Conclusion:**

This quantitative deficiency in CD8^+^ T cell immunity to EBV might be responsible for the accumulation of EBV infected B cells in the brains of patients with MS.

A large body of evidence indicates that multiple sclerosis (MS) is an autoimmune disease^1^ ^2^ but the primary cause of MS and the other human chronic autoimmune diseases is as yet unknown. Epidemiological studies indicate that infection with the Epstein–Barr virus (EBV) has a role in the pathogenesis of MS.^3^ In a meta-analysis of 13 case control studies comparing EBV serology in patients with MS and controls, 99.5% of patients with MS were EBV seropositive compared with 94.0% of controls, with EBV seronegativity having an OR_MH_ odds ratio of MS of 0.06 (exact 95% confidence interval 0.03 to 0.13; p<0.000000001).^3^ Furthermore, children with MS have an EBV seropositivity rate of 98.6% compared with 72.1% in age matched controls.^4^ These studies suggest that EBV infection is a prerequisite for the development of MS.

EBV has the unique ability to infect, activate and latently persist in B lymphocytes. When EBV infects resting B cells, it drives them into activation and proliferation independently of T cell help. Usually, the proliferating infected B cells are eventually eliminated by EBV specific cytotoxic CD8^+^ T cells but latently infected non-proliferating memory B cells persist in the individual for life.^5^ We have hypothesised that a genetically determined defect in the elimination of EBV infected B cells by cytotoxic CD8^+^ T cells might predispose to the development of MS by allowing the accumulation of EBV infected autoreactive B cells in the CNS.^6^ Recently it has been demonstrated that a substantial proportion of the B cells and plasma cells in the MS brain are infected with EBV.^7^

Several studies have investigated T cell immunity to EBV in MS and have yielded conflicting results. Craig and colleagues^8^ found that patients with MS have decreased T cell control of the number of immunoglobulin (Ig) secreting B cells after in vitro infection with EBV. Using a panel of five HLA-A2 restricted EBV peptides and one HLA-B7 restricted EBV peptide, Höllsberg and colleagues^9^ found an increased frequency of CD8^+^ T cells reactive to two immunodominant EBV epitopes in patients with MS. However, another study^10^ found no differences in the CD8^+^ T cell frequencies between patients with MS and healthy controls for seven HLA-B7 restricted EBV peptides, including one of the peptides found by Höllsberg and colleagues^9^ to elicit an increased response. Using a pool of 18 HLA class I restricted EBV peptides, Jilek and colleagues^11^ found an increased EBV specific CD8^+^ T cell response in patients with clinically isolated syndromes but a normal response in established MS whereas another study found that patients with MS had an increased CD4^+^ T cell response to peptides derived from EBV nuclear antigen-1 (EBNA1).^12^

Studies on T cell immunity using selected EBV peptides are limited by the fact that they do not provide a measure of the total T cell response to EBV, which encodes many different proteins. In the present study we have investigated T cell immunity to EBV in MS, focusing on the total T cell response to EBV infected B cells using EBV infected B cell lymphoblastoid cell lines (LCL) as stimulators. LCL express not only the latent proteins of EBV but also the lytic proteins,^13^ ^14^ owing to the fact that a proportion of the cells in LCL are in the lytic phase of infection. Our specific aims were to determine: (1) whether the frequency of T cells specific for EBV infected B cells is increased or decreased in patients with MS; (2) whether EBV infected B cells of patients with MS can be killed by cytotoxic CD8^+^ T cells from HLA matched healthy subjects; (3) whether EBV specific CD8^+^ T cells generated in vitro from patients with MS can kill EBV infected B cells; and (4) whether there is a correlation between T cell and antibody immunity to EBV.

## METHODS

### Subjects

Blood (60 ml) was collected from 34 EBV seropositive healthy subjects and 34 EBV seropositive patients with MS following informed consent. This study was approved by the Royal Brisbane and Women’s Hospital Human Research Ethics Committee and the University of Queensland Medical Research Ethics Committee. All patients met the 2005 Revised McDonald criteria for a diagnosis of MS.^15^ The clinical course was relapsing–remitting in 14 patients, secondary progressive in 11 and primary progressive in nine. Patients had not received corticosteroids or immunomodulatory therapy for at least 3 months prior to venesection. Only five of the patients had ever received interferon β, glatiramer acetate or immunosuppressant drugs, and in three of these the treatment had been stopped at least 2 years before blood was collected for this study. Disability was assessed using the Kurtzke Expanded Disability Status Scale (EDSS)^16^ and the MS Severity Score (MSSS) was determined from the EDSS and disease duration.^17^ The demographic and clinical details of the healthy subjects and patients with MS are presented in table 1. Subjects were Caucasian except for one healthy subject and two patients with MS who were Asian.

**Table 1:** Characteristics of healthy subjects and patients with MS

	HC	MS
n	34	34
Female sex (n)	26	19
Mean age (years)	39.5 (9.9)	43.8 (11.8)
Mean age of onset of MS (years)		34.1 (10.9)
Mean duration of MS (years)		10.1 (7.6)
Mean EDSS		4.8 (2.4)
Mean MSSS		6.2 (3.0)

EDSS, Expanded Disability Status Scale; HC, healthy control subjects; MS, multiple sclerosis; MSSS, MS Severity Score.

### Processing of blood samples

Blood (10 ml) was used for DNA extraction and HLA typing, and 5 ml for serum collection. Peripheral blood mononuclear cells (PBMC) were separated by density centrifugation, resuspended in complete RPMI (RPMI-C) with 10% dimethyl sulphoxide (Sigma, St Louis, Missouri, USA) and cryopreserved in liquid nitrogen. RPMI-C was prepared by supplementing RPMI-1640 tissue culture medium (Invitrogen, Carlsbad, California, USA) with 10% heat inactivated fetal calf serum (Cambrex, Walkersville, Maryland, USA), 2 mM L-glutamine (Lonza, Walkersville, Maryland, USA), 50 U/ml penicillin (Invitrogen), 50 μg/ml streptomycin (Invitrogen) and 0.01 M HEPES buffer (Lonza, Walkersville, Maryland, USA).

### Antiviral antibodies

Serum samples were diluted in doubling dilutions ranging from 1/12.5 to 1/12800 and tested using enzyme linked immunosorbent assay (ELISA) kits (Inverness Medical, Brisbane, Australia) to determine the titres of IgG specific for EBNA1 and EBV viral capsid antigen (VCA).

### HLA typing

Genomic DNA was extracted from 10 ml of heparinised blood using NucleoSpin Blood XL DNA extraction kits (Macherey-Nagel, Düren, Germany). Low resolution sequence specific primer kits (Dynal Biotech, Oslo, Norway) were used to type for HLA-A, HLA-B, HLA-DR, HLA-DQA and HLA-DQB.

### Generation of LCL and assessment by flow cytometry

LCL were generated from each subject by incubating 50 000 washed PBMC with the B95-8 strain of EBV overnight in 1 ml of RPMI-C supplemented with 2 μg/ml ciclosporin (Sigma). LCL were cultured in this medium for 1 month followed by at least another 2 months of culture in RPMI-C without ciclosporin until confluent rapidly growing lines were obtained. These LCL were flow cytometrically assessed after 3 months of culture to confirm that they contained only CD19^+^ B cells, with no contaminating CD3^+^ T cells, before being used in ELISPOT assays, cytotoxicity assays or for the generation of LCL specific T cell lines. To quantify HLA class I expression by LCL, healthy log phase cultures of LCL were stained with R-PE labelled antihuman HLA-ABC (BD Pharmingen, San Diego, California, USA).

### ELISPOT assays

To measure the total T cell response to EBV infected B cells, we incubated PBMC with autologous LCL. PBMC were thawed from liquid nitrogen storage and incubated in RPMI-C for 24 h before use to allow re-expression of cell surface receptors. After counting and assessment for viability by trypan blue staining, PBMC were cultured in quadruplicate with either 50 000 or 5000 cells per well in 200 μl volumes of RPMI-C in the presence of 50 000 autologous LCL or in the absence of LCL for 48 h. Interferon γ (IFNγ) enzyme linked immunospot (ELISPOT) assays were conducted using a commercially available kit (Mabtech, Stockholm, Sweden), according to the manufacturer’s instructions. The average number of IFNγ spot forming cells (SFC) per well in the presence of PBMC and absence of LCL, and the average number of SFC per well in the presence of LCL and absence of PBMC were added together for each subject. The sum of these backgrounds was subtracted from the average number of SFC per well in the presence of both PBMC and LCL, with the difference expressed as the number of LCL specific IFNγ SFC per 10^6^ PBMC. The mean background for PBMC alone was 7 (7) SFC per 10^6^ cells in healthy subjects and 13 (19) SFC per 10^6^ cells in patients with MS, and the mean background for LCL alone was 33 (53) SFC per 10^6^ cells in healthy subjects and 73 (90) SFC per 10^6^ cells in patients with MS. In some subjects we also used the IFNγ ELISPOT assay to measure the frequency of PBMC specific for synthetic EBV peptides by incubating 200 000 PBMC per well with and without 10 μg/ml of each peptide.

### T cell cytotoxicity assays

Chromium release assays were performed to measure the killing of LCL by a panel of five non-autologous HLA matched EBV specific CD8^+^ T cell clones derived from healthy subjects in previous studies by SRB.^18^ LCL were washed, labelled for 60 min with 3.7×10^6^ Bq of ^51^Cr (Na_2_Cr_3_O_4_) at 37°C and then washed twice with medium. The ^51^Cr-labelled target cells (10^4^ per well) were mixed with T cells from HLA matched T cell clones at effector:target ratios of 4:1 and 2:1 in 96 well round bottomed plates in 200 μl volumes. After 5 h of incubation, 25 μl of supernatant from each well were counted in a β scintillation counter (Packard Instrument, Meriden, Connecticut, USA). Maximum release and spontaneous release of ^51^Cr were measured in wells containing target cells in the presence of 10% sodium dodecyl sulphate or medium alone, respectively. The per cent specific lysis was calculated by the formula:

% specific lysis  =  (test release − spontaneous release)/(maximum release − spontaneous release) × 100.

### Generation and testing of EBV specific T cell lines

To generate EBV specific T cell lines, PBMC were incubated with 10^6^ irradiated (80 Gy) autologous LCL (stimulator to responder ratio of 1:10) in RPMI-C without interleukin 2 (IL2) in 24 well tissue culture plates. Eleven days later, each cell line was restimulated with irradiated autologous LCL, and from day 13 the medium was supplemented with 20 U/ml recombinant IL2. A third stimulation with irradiated autologous LCL was given on day 22. Cultures were immunophenotyped by flow cytometry and tested for cytotoxic capacity at day 40. The T cell lines were assessed by flow cytometry to determine the proportions of CD4^+^CD3^+^ T cells, CD8^+^CD3^+^ T cells and CD16^+^CD3^−^/CD56^+^CD3^−^ NK cells using directly conjugated antibodies (BD Pharmingen, San Diego, California, USA). The cytotoxic capacity of the T cell lines was determined by 5 h chromium release assays using autologous LCL and HLA mismatched LCL as targets, with effector:target ratios of 20:1, 10:1, 5:1 and 2.5:1.

### Statistical analysis

Statistical analyses were performed using SigmaStat 3.11.0 (Systat Software, Inc., San Jose, California, USA). For comparisons between patients with MS and healthy subjects when the data were normally distributed, the means were compared using the Student’s t test. If the data were not normally distributed, the medians were compared using the Mann–Whitney rank sum test. Differences between means or between medians were considered significant if p⩽0.05. Means are presented as mean (SD). To compare the proportions of subjects with each titre of anti-EBNA1 or anti-VCA IgG, we used the χ^2^ test with Yates’ correction as required. To measure the correlation between data, the parametric Pearson product moment correlation was used for normally distributed data, and the Spearman rank order correlation was used for non-parametric data.

## RESULTS

### Patients with MS have decreased T cell immunity to EBV

In preliminary studies we used ELISPOT assays to determine the frequency of PBMC producing IFNγ in response to synthetic peptides derived from a variety of EBV proteins and restricted by HLA molecules commonly carried by patients with MS. When analysing these experiments, three problems became apparent. Firstly, the T cell responses to any given EBV peptide could be compared only between those subjects who carried the restricting HLA molecule. Secondly, the use of selected EBV peptides did not provide a measure of the total T cell response to EBV in any subject. Thirdly, the use of peptides bypasses the normal physiological process of antigen processing. Thus a person might have a high frequency of T cells producing IFNγ in response to an exogenously added synthetic EBV peptide which is presented at a low density on the surface of EBV infected B cells so that the infected B cells are poorly recognised by peptide specific T cells.^19^

**Figure 1:**
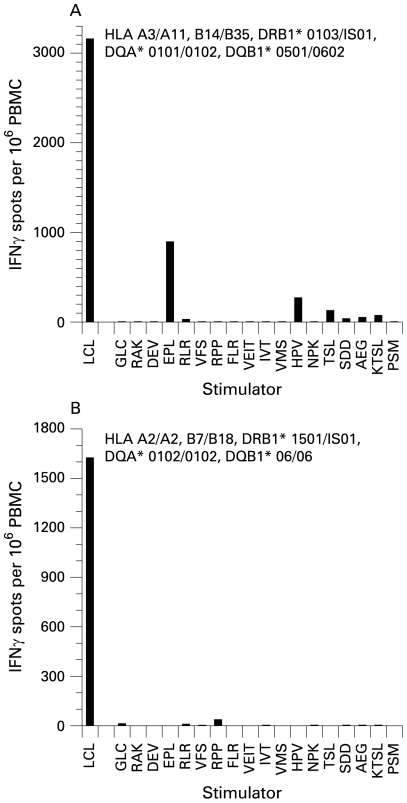
Frequencies of T cells producing interferon γ (IFNγ) in response to autologous lymphoblastoid cell lines (LCL) and to individual synthetic peptides derived from Epstein–Barr virus (EBV) proteins in an EBV seropositive healthy subject (A) and an EBV seropositive multiple sclerosis (MS) patient (B). Each peptide is represented by the first three amino acids of its sequence. In the healthy subject (A), the highest response to any peptide was against EPLPQGQLTAY, derived from the EBV lytic cycle protein BZLF1 and restricted by HLA-B35, which was carried by this subject. The next highest peptide response was against HPVGEADYFEY, derived from the latent cycle protein EBNA1 and also restricted by HLA-B35. The third highest response was against TSLYNLRRGTALAI, derived from EBNA1 and restricted by HLA-DR1, which was carried by this subject. The other peptides, proteins from which they are derived and their HLA restriction elements are as follows: GLC, GLCTLVAML from BMLF1, HLA-A2; RAK, RAKFKQLL from BZLF1, HLA-B8; DEV, DEVEPLGHY from BMLF1, HLA-B18; RLR, RLRAEAQVK from EBNA3A, HLA-A3; VFS, VFSDGRVAC from EBNA3A, HLA-A29; RPP, RPPIFIRRL from EBNA3A, HLA-B7; FLR, FLRGRAYGL from EBNA3A, HLA-B8; VEIT, VEITPYKPTW from EBNA3B, HLA-B44; IVT, IVTDFSVIK from EBNA3B, HLA-A11; VMS, VMSNTLLSAW from latent membrane protein 2, HLA-A25; NPK, NPKFENIAEGLRALL from EBNA1, HLA-DR11; SDD, SDDELPYIDPNMEPV from EBNA3C, HLA-DR11; AEG, AEGLRALLARSHVER from EBNA1, HLA-DR15; KTSL, KTSLTNLRRGTALA from EBNA1, HLA-DR7; and PSM, PSMPFASDYSQGAFT from EBNA3C, HLA-DR4. In the patient with MS (B), the highest response to any peptide was against RPPIFIRRL, derived from the EBV latent cycle protein EBNA3A and restricted by HLA-B7, which was carried by this patient. In both subjects the sum of the responses to the 18 individual peptides tested was much lower than the response to the LCL.

To overcome these problems, we measured the frequency of PBMC producing IFNγ in response to autologous LCL. This approach provides a direct measure of the aggregate T cell response to EBV infected B cells in each subject and also allows comparisons between subjects regardless of which HLA genes are carried. Figure 1 shows that the sum of the responses to the 18 individual synthetic peptides tested is much lower than the response to the LCL in a given subject. By extracting purified populations of CD4^+^ T cells, CD8^+^ T cells and NK cells from PBMC using immunomagnetic beads, we found that LCL specific T cells were 5–7-fold more frequent in the CD8^+^ T cell population than in the CD4^+^ T cell population and less frequent again in the NK cell population (not shown).

**Figure 2:**
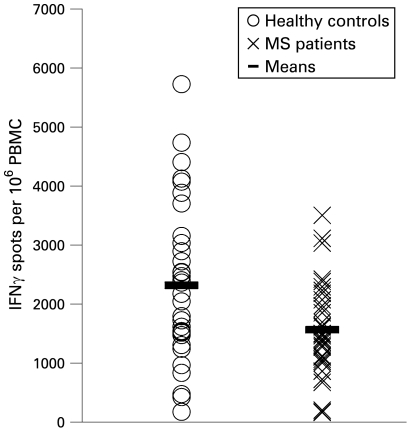
Frequencies of peripheral blood mononuclear cell (PBMC) producing interferon γ (IFNγ) in response to autologous lymphoblastoid cell lines (LCL) in 34 Epstein–Barr virus (EBV) seropositive healthy subjects and 34 EBV seropositive patients with multiple sclerosis (MS). The mean frequency in patients with MS (1573 (798) per 10^6^ PBMC) was significantly lower than the mean in healthy subjects (2326 (1314) per 10^6^ PBMC) (p = 0.006, heteroscedastic t test).

We then measured the T cell reactivity against LCL in 34 EBV seropositive healthy subjects and 34 EBV seropositive patients with MS (fig 2). The specificity of this response as a measure of EBV immunity was demonstrated by the fact that the frequency in an EBV seronegative healthy subject was only 80 per 10^6^ PBMC, which was much lower than the mean in EBV seropositive healthy subjects. The mean LCL specific T cell frequency in the EBV seropositive patients with MS was 1573 (798) per 10^6^ PBMC, which was significantly lower than that in EBV seropositive healthy subjects (2326 (1314) per 10^6^ PBMC; p = 0.006) (fig 2). The mean LCL specific T cell frequency per 10^6^ PBMC was 1635 (898) in patients with relapsing–remitting MS, 1235 (437) in patients with secondary progressive MS and 1888 (897) in patients with primary progressive MS. The frequency in female patients (1551 (900) per 10^6^ PBMC) was similar to that in male patients (1600 (677) per 10^6^ PBMC; p = 0.86). There were no significant correlations between the LCL specific T cell frequency and patient age, disease duration, EDSS or MSSS. Interestingly, there was a significant correlation between the T cell frequency and age of onset of MS (r = 0.38; p = 0.028)—that is, the lower the T cell frequency the earlier the onset of MS.

These results indicate that patients with MS have a decreased T cell response to EBV infected B cells. Because EBV specific T cell IFNγ production correlates with EBV specific T cell cytotoxicity,^20^ this suggests that patients with MS have a defective ability to eliminate EBV infected B cells. One possible explanation for decreased CD8^+^ T cell reactivity to LCL is decreased HLA class I expression by LCL. The mean geometric mean fluorescence intensity of HLA class I on LCL was 434 (173) in healthy subjects and 425 (173) in patients with MS (p = 0.82), showing that the decreased T cell reactivity to LCL in patients with MS was not due to decreased HLA class I expression on LCL and was most likely due to an absolute decrease in the numbers of CD8^+^ T cells reacting to LCL.

### Decreased T cell immunity to EBV in MS is not explained by differences in HLA genes

The results of HLA-A, HLA-B, HLA-DRB, HLA-DQA and HLA-DQB typing are presented in table 2. The main differences between patients with MS and healthy subjects were as follows: patients with MS carried A*02 and B*44 less frequently, and B*07 and DRB1*15 more frequently, than healthy subjects, as previously described.^21^ There was no significant difference in the frequency of T cells producing IFNγ against LCL between HLA-A2^+^ and HLA-A2^–^ healthy subjects, between HLA-A2^+^ and HLA-A2^−^ patients with MS, between HLA-B7^+^ and HLA-B7^−^ healthy subjects, between HLA-B7^+^ and HLA-B7^−^ patients with MS, between HLA-B44^+^ and HLA-B44^−^ healthy subjects, between HLA-B44^+^ and HLA-B44^−^ patients with MS, between HLA-DR15^+^ and HLA-DR15^−^ healthy subjects or between HLA-DR15^+^ and HLA-DR15^−^ patients with MS, indicating that differences in HLA genes carried by patients with MS and healthy subjects do not account for the decreased T cell response to LCL in patients with MS.

**Table 2:** HLA class I and class II typing

HLA class I			HLA class II		
HLA allele	HC (n (%))†	MS (n (%))†	HLA allele	HC (n (%))	MS (n (%))
A*01	9 (26.5)	6 (17.6)	DRB1*01	7 (20.6)	7 (20.6)
A*02	16 (47.1)	11 (32.4)	DRB1*15	12 (35.3)	18 (52.9)
A*03	10 (29.4)	11 (32.4)	DRB1*16	0 (0.0)	2 (5.9)
A*11	4 (11.8)	7 (20.6)	DRB1*03	9 (26.5)	7 (20.6)
A*23	5 (14.7)	1 (2.9)	DRB1*04	13 (38.2)	6 (17.6)
A*24	2 (5.9)	6 (17.6)	DRB1*11	5 (14.7)	3 (8.8)
A*25	2 (5.9)	2 (5.9)	DRB1*12	1 (2.9)	2 (5.9)
A*26	1 (2.9)	0 (0.0)	DRB1*13	7 (20.6)	5 (14.7)
A*29	2 (5.9)	0 (0.0)	DRB1*14	2 (5.9)	0 (0.0)
A*30	1 (2.9)	1 (2.9)	DRB1*07	8 (23.5)	6 (17.6)
A*31	0 (0.0)	3 (8.8)	DRB1*08	2 (5.9)	2 (5.9)
A*32	2 (5.9)	3 (8.8)	DRB1*09	0 (0.0)	0 (0.0)
A*33	1 (2.9)	1 (2.9)	DRB1*10	1 (2.9)	2 (5.9)
A*36	0 (0.0)	2 (5.9)			
A*68	1 (2.9)	2 (5.9)	DQA1*01	26 (76.5)	28 (82.4)
A*74	1 (2.9)	0 (0.0)	DQA1*02	8 (23.5)	7 (20.6)
A*92	1 (2.9)	0 (0.0)	DQA1*03	14 (41.2)	6 (17.6)
			DQA1*04	2 (5.9)	3 (8.8)
B*07	7 (20.6)	14 (41.2)	DQA1*05	14 (41.2)	11 (32.4)
B*08	7 (20.6)	6 (17.6)			
B*13	4 (11.8)	3 (8.8)	DQB1*02	15 (44.1)	11 (32.4)
B*14	4 (11.8)	2 (5.9)	DQB1*03	17 (50.0)	13 (38.2)
B*15	4 (11.8)	2 (5.9)	DQB1*04	1 (2.9)	2 (5.9)
B*18	2 (5.9)	4 (11.8)	DQB1*05	10 (29.4)	11 (32.4)
B*27	2 (5.9)	1 (2.9)	DQB1*06	18 (52.9)	21 (61.8)
B*35	4 (11.8)	8 (23.5)			
B*37	2 (5.9)	1 (2.9)			
B*38	1 (2.9)	1 (2.9)			
B*39	1 (2.9)	4 (11.8)			
B*40	4 (11.8)	4 (11.8)			
B*44	12 (35.3)	6 (17.6)			
B*49	2 (5.9)	0 (0.0)			
B*51	4 (11.8)	1 (2.9)			
B*52	1 (2.9)	1 (2.9)			
B*53	0 (0.0)	1 (2.9)			
B*55	2 (5.9)	2 (5.9)			
B*57	1 (2.9)	4 (11.8)			

†Number (%) of subjects carrying each allele, either heterozygously or homozygously.

HC, 34 healthy control subjects; MS, 34 patients with multiple sclerosis.

### EBV infected B cells from patients with MS are normally susceptible to killing by cytotoxic CD8^+^ T cells

To determine whether EBV infected B cells from patients with MS can be killed by EBV specific CD8^+^ T cells, we used chromium release assays to measure the killing of LCL by HLA matched EBV specific CD8^+^ T cell clones derived from healthy subjects (table 3). Using a CD8^+^ T cell clone specific for the HLA-A3 restricted RLRAEAQVK peptide of EBNA3A,^22^ we found that the killing of LCL from HLA-A3^+^ patients with MS and healthy subjects was similar. With a CD8^+^ T cell clone specific for the HLA-B7 restricted RPPIFIRRL peptide of EBNA3A,^22^ the killing of LCL from HLA-B7^+^ patients with MS and healthy subjects was also similar. With a CD8^+^ T cell clone specific for the HLA-A11 restricted AVFDRKSDAK peptide of EBNA3B,^23^ the killing of LCL from HLA-A11^+^ patients with MS and healthy subjects was also similar. These results indicate that EBV infected B cells from patients with MS can be killed by EBV specific CD8^+^ T cells just as readily as those from healthy subjects. The results are also consistent with our finding of normal HLA class I expression by LCL in patients with MS and provide further evidence that the decreased T cell reactivity to LCL is not due to an LCL abnormality.

**Table 3:** Susceptibility of LCL to killing by HLA matched EBV specific CD8^+^ T cell clones from healthy subjects

Subject	Per cent specific lysis of LCL by T cell clone					
	HLA-A3* RLRAEAQVK† clone JC/RLR2		HLA-B7 RPPIFIRRL clone JO/RPPI7§		HLA-A11 AVFDRKSDAK clone CM/AVF29§	
	E:T = 4:1	E:T = 2:1	E:T = 4:1	E:T = 2:1	E:T = 4:1	E:T = 2:1
HC 2	42.3	30.2				
HC 5			5.4	4.4		
HC 9			17.3	15.2		
HC 11	46.2	34.1	21.0	16.9		
HC 14	62.8	49.5				
HC 15	26.1	23.1	20.4	18.2		
HC 16	37.2	29.5			49.4	50.7
HC 21	38.3	31.6	9.9	9.7		
HC 25					31.4	31.5
HC 26	40.3	32.2				
HC 30			9.4	4.9		
HC 34					26.0	27.5
Mean HC	41.9 (11.1)	32.9 (8.1)	13.9 (6.5)	11.6 (6.1)	35.6 (12.3)	36.6 (12.4)
MS 143	62.8	47.2	31.5	25.1		
MS 187	71.3	60.3	11.8	10.1		
MS 188			25.4	21.8	53.7	52.2
MS 196	39.9	31.5	16.3	15.8		
MS 200	37.5	33.8	8.5	6.5		
MS 201	57.1	38.0				
MS 212			13.7	12.8		
MS 215			11.1	11.1	31.1	30.9
MS 329			4.4	1.6	12.6	14.6
MS 419	33.9	30.7	10.1	10.2		
MS 442	45.9	35.8				
MS 468	49.5	42.0				
Mean MS	49.7 (13.1)	39.9 (9.9)	14.8 (8.6)	12.8 (7.3)	32.5 (20.6)	32.6 (18.9)
p Value‡	0.24	0.16	0.84	0.74	0.83	0.77

*HLA restriction element of T cell clone.

†EBV peptide recognised by T cell clone.

‡Student’s t test for comparison between mean HC and mean MS.

§Similar results were obtained using another CD8^+^ T cell clone specific for the same peptide.

EBV, Epstein–Barr virus; E:T, effector:target ratio; LCL, lymphoblastoid cell lines; Mean HC, mean (SD) in healthy control subjects; Mean MS, mean (SD) in patients with multiple sclerosis.

### Patients with MS can generate normal EBV specific cytotoxic CD8^+^ T cell lines in vitro

One possible explanation for the decreased frequency of LCL specific T cells in patients with MS is a primary T cell defect. To investigate this, we generated T cell lines from 10 healthy subjects and 11 patients with MS by stimulating PBMC with irradiated autologous LCL. The T cell lines from six healthy subjects and six patients with MS specifically killed autologous LCL but not HLA mismatched LCL (table 4). The EBV specific T cell lines were composed predominantly of CD8^+^ T cells in most subjects. When tested against autologous LCL, the cytotoxic capacity of the T cell lines was similar in healthy subjects and patients with MS. These results indicate that, despite a lower frequency of EBV specific T cells in patients with MS, EBV specific cytotoxic CD8^+^ T cell lines can be generated normally in vitro from these patients. Thus the decreased frequency of circulating LCL specific T cells cannot be explained by a primary T cell defect.

**Table 4:** Phenotype and cytotoxicity of LCL specific T cell lines

Subject	Phenotype of T cell line (%)			Per cent specific lysis of LCL			
				Autologous LCL		HLA mismatched LCL	
	CD4^+^	CD8^+^	NK	E:T = 20:1	E:T = 10:1	E:T = 20:1	E:T = 10:1
HC 4	7.7	86.9	0.9	46.5	37.4	13.4	8.1
HC 12	16.1	85.3	1.1	40.2	36.2	6.3	6.0
HC 14	59.4	31.7	0.6	36.8	27.1	10.1	6.5
HC 24	29.5	67.2	2.8	54.3	49.4	19.1	15.1
HC 31	23.4	71.9	3.8	52.4	43.1	9.1	7.6
HC 32	47.8	45.2	0.7	49.0	40.9	6.1	5.5
Mean HC	30.6 (19.6)	64.7 (22.1)	1.0*	46.5 (6.9)	39.0 (7.5)		
MS 192	60.9	38.8	1.0	17.0	7.1	−1.7	−5.1
MS 210	20.3	73.2	3.5	69.2	63.3	12.1	9.1
MS 211	45.3	54.8	0.5	39.3	29.5	2.3	3.7
MS 212	7.6	90.0	1.7	45.5	30.1	9.0	5.4
MS 215	40.5	60.1	1.1	46.3	35.4	5.4	2.1
MS 428	19.0	63.3	8.7	95.1	96.2	15.9	14.9
Mean MS	32.3 (19.9)	63.4 (17.3)	1.4*	52.1 (26.9)	43.6 (31.4)		
p Value†	0.89	0.91	0.70‡	0.64	0.74		

*Median.

†Student’s t test for comparison between Mean HC and Mean MS.

‡Mann–Whitney rank sum test for comparison of median per cent NK cells between HC and MS.

E:T, effector:target ratio; LCL, lymphoblastoid cell lines; Mean HC, mean (SD) in healthy control subjects; Mean MS, mean (SD) in patients with multiple sclerosis.

### Correlation between T cell and antibody immunity to EBV

Levels of anti-EBNA1 IgG and anti-VCA IgG were quantified by titration of serum samples using ELISA (table 5). The geometric mean titre of anti-EBNA1 IgG was higher in patients with MS (1155) than in healthy subjects (720). The proportion of individuals with a high titre (⩾1600) of anti-EBNA1 IgG was also higher in the MS group than in the healthy control group although the difference was not significant. The median absorbance index for anti-EBNA1 IgG at a dilution of 1:200 was significantly higher in patients with MS (2.33) than in healthy subjects (2.13; p = 0.025). There was an inverse correlation between the anti-EBNA1 IgG titre and the LCL specific T cell frequency (r_s_ = −0.22, p = 0.069) when the results from healthy subjects and patients with MS were combined. The geometric mean titre of anti-VCA IgG was similar in patients with MS and healthy subjects, as was the proportion of individuals with a titre ⩾1600. There was no correlation between the anti-VCA IgG titre and the LCL specific T cell frequency.

**Table 5:** Titres of anti-EBNA1 IgG and anti-VCA IgG in serum

Titre	Anti-EBNA1 IgG		Anti-VCA IgG	
	HC (n (%))	MS (n (%))	HC (n (%))	MS (n (%))
50	2 (6.1)	0 (0.0)	1 (3.0)	0 (0.0)
100	1 (3.0)	0 (0.0)	2 (6.1)	0 (0.0)
200	5 (15.2)	3 (8.8)	1 (3.0)	1 (2.9)
400	6 (18.2)	6 (17.6)	0 (0.0)	4 (11.8)
800	7 (21.2)	7 (20.6)	5 (15.2)	4 (11.8)
1600	6 (18.2)	10 (29.4)	9 (27.3)	15 (44.1)
3200	2 (6.1)	4 (11.8)	14 (42.4)	8 (23.5)
6400	4 (12.1)	4 (11.8)	1 (3.0)	1 (2.9)
12800	0 (0.0)	0 (0.0)	0 (0.0)	1 (2.9)
GMT	720	1155	1440	1536
n (%) titre ⩾1600	12 (36.4)	18 (52.9)	24 (72.7)	25 (73.5)
p Value*		0.17		0.94

*χ^2^ test comparing proportions of HC and MS subjects with titre ⩾1600.

EBNA1, Epstein–Barr virus nuclear antigen 1; GMT, geometric mean titre; HC, 33 healthy control subjects; MS, 34 patients with multiple sclerosis; VCA, viral capsid antigen.

## DISCUSSION

In this study, we have shown that patients with MS have decreased T cell immunity to EBV, as assessed by the frequency of PBMC producing IFNγ in response to autologous LCL. In contrast to the use of synthetic EBV peptides as target antigens, this approach provides a direct measure of the aggregate T cell response to EBV infected B cells in each subject because it uses each person’s natural antigen processing mechanisms to present viral antigens at normal physiological concentrations on the surface of their own EBV infected B cells and it represents the total T cell response to all EBV antigens presented by all HLA molecules on LCL in each subject. Furthermore, LCL stimulation detects responses not only to the latent proteins of EBV but also to the lytic proteins.^13^ ^14^ Our finding of decreased T cell immunity to EBV in MS is not a reflection of a generalised depression in T cell immunity because patients with MS have normal T cell responses to tetanus toxoid and concanavalin A and increased responses to myelin antigens.^24^

By studying the T cell response to LCL in purified populations of CD4^+^ T cells, CD8^+^ T cells and NK cells, we have demonstrated that CD8^+^ T cells are the predominant population responding to EBV infected B cells, with the frequency of EBV specific CD8^+^ T cells being 5–7-fold higher than the frequency of EBV specific CD4^+^ T cells, as previously reported.^20^

Our finding of decreased T cell immunity to LCL in MS differs from a previous small study on 11 patients which reported a non-significant increase in the frequency of LCL specific CD8^+^ T cells, as determined by intracellular cytokine flow cytometry.^25^ The most likely explanation for this discrepancy is the small sample size of that study. Our results are consistent with an early study reporting that patients with MS have decreased T cell control of the number of Ig secreting B cells after in vitro infection with EBV.^8^ A recent study using a pool of 18 HLA class I restricted EBV peptides in ELISPOT assays found an increased EBV specific CD8^+^ T cell response in patients with clinically isolated syndromes but a normal response in established MS.^11^ The results of that study are difficult to interpret because it is likely that in any given subject only a minority of the peptides in the peptide pool were restricted by HLA class I alleles carried by the subject. Thus the T cell response to the pool of peptides might have been composed largely of responses that have no in vivo relevance. It is noteworthy that the mean frequency of T cells responding to the pool of peptides in healthy subjects in that study was only 5% of the mean frequency of LCL specific T cells in healthy subjects in our study.

We have shown that the decreased CD8^+^ T cell response to LCL in MS is not due to decreased HLA class I expression on the LCL and that LCL from patients with MS can be killed normally by HLA matched EBV specific cytotoxic CD8^+^ T cell clones from healthy subjects. We have also shown that differences in HLA genes carried by patients with MS and healthy subjects do not account for the decreased T cell response to LCL in MS. In addition, we have demonstrated that the decreased CD8^+^ T cell response to EBV infected B cells is not caused by a primary defect in the function of CD8^+^ T cells because EBV specific cytotoxic CD8^+^ T cell lines could be generated normally in vitro from PBMC of patients with MS. One possible explanation for the decreased CD8^+^ T cell immunity to EBV in MS is impaired priming of EBV specific CD8^+^ T cells by dendritic cells. A limitation of our study is that we have not determined whether the decrease in CD8^+^ T cell immunity to EBV is accompanied by a decrease in CD8^+^ T cell immunity to other infectious agents. Further studies will be required to investigate this.

Our finding of an elevated anti-EBNA1 IgG titre in patients with MS is consistent with previous reports.^25^^–^27 We also observed an inverse correlation between the anti-EBNA1 IgG titre and the anti-LCL T cell frequency, suggesting that the elevated anti-EBNA1 IgG titre might be a consequence of decreased CD8^+^ T cell immunity to EBV. Prospective studies of blood samples collected before the onset of MS have shown that elevated IgG reactivity to EBNA1 increases the risk of developing MS.^28^^–^30

A fundamental question is whether the decreased frequency of EBV specific T cells in the blood is the cause or effect of the accumulation of EBV infected B cells in the CNS in MS,^7^ or a combination of both. Some of the decrease might be due to sequestration of EBV specific CD8^+^ T cells in the CNS. CD8^+^ T cells have been shown to be present in the CNS in proportion to the number of EBV infected B cells^7^ but it is not known whether these CD8^+^ T cells are specific for EBV and whether they can recognise and kill EBV infected B cells in the CNS. If the decreased frequency of LCL specific T cells in the blood were to be attributed solely to sequestration within the CNS, it would beg the question why EBV infected B cells accumulate in the CNS in the first place and why are they not eliminated by EBV specific CD8^+^ T cells, given that we have shown that EBV infected B cells of patients with MS can be killed by their own EBV specific CD8^+^ T cells. We suggest that the most likely scenario is that a genetically determined quantitative deficiency in the generation of EBV specific CD8^+^ T cells in vivo allows the accumulation of EBV infected B cells in the CNS in MS and that a vicious circle then ensues whereby the inherently deficient CD8^+^ T cell response is further compromised by T cell exhaustion^31^ as a result of the persistent high EBV load in the CNS. It could be argued that the decrease in EBV specific CD8^+^ T cell immunity in patients with MS reported here is of an insufficient degree to lead to the development of MS. However, in non-linear dynamic systems, such as those commonly occurring in biology and medicine, small changes in the initial condition of a system can produce dramatic changes in long term behaviour, sometimes referred to as the “butterfly effect”.^32^ ^33^ Our finding that lower LCL specific T cell frequencies were associated with earlier age of onset of MS suggests that the more severe the defect in EBV specific T cell immunity the sooner MS will develop after primary EBV infection. EBV specific CD8^+^ T cells can also be sequestered in the bone marrow^34^ but there is no reason to believe that this should occur to a greater extent in patients with MS than in healthy subjects.

In conclusion, the quantitative deficiency in CD8^+^ T cell immunity to EBV reported in the present study might predispose to the development of MS by allowing the accumulation of EBV infected autoreactive B cells in the CNS.^6^ Boosting CD8^+^ T cell immunity to EBV by vaccination or by transferring EBV specific cytotoxic CD8^+^ T cells may be beneficial in preventing and treating MS.
